# Race and Drug Toxicity: A Study of Three Cardiovascular Drugs with Strong Pharmacogenetic Recommendations

**DOI:** 10.3390/jpm11111226

**Published:** 2021-11-18

**Authors:** Travis J. O’Brien, Kevin Fenton, Alfateh Sidahmed, April Barbour, Arthur F. Harralson

**Affiliations:** 1 Department of Pharmacology and Physiology, George Washington University, Washington, DC 20052, USA; 2 Department of Biostatistics, George Washington University, Washington, DC 20052, USA; kfenton19@gwmail.gwu.edu; 3 Department of Medicine, George Washington University, Washington, DC 20052, USA; asidahmed@mfa.gwu.edu (A.S.); abarbour@mfa.gwu.edu (A.B.); 4 Department of Pharmacogenomics, Bernard J. Dunn School of Pharmacy, Shenandoah University, Winchester, VA 22601, USA; aharrals@su.edu

**Keywords:** pharmacogenomics, clopidogrel, warfarin, simvastatin, race, ethnicity, toxicity

## Abstract

The Clinical Pharmacogenetics Implementation Consortium (CPIC^®^) establishes evidence-based guidelines for utilizing pharmacogenetic information for certain priority drugs. Warfarin, clopidogrel and simvastatin are cardiovascular drugs that carry strong prescribing guidance by CPIC. The respective pharmacogenes for each of these drugs exhibit considerable variability amongst different ethnic/ancestral/racial populations. Race and ethnicity are commonly employed as surrogate biomarkers in clinical practice and can be found in many prescribing guidelines. This is controversial due to the large variability that exists amongst different racial/ethnic groups, lack of detailed ethnic information and the broad geographic categorization of racial groups. Using a retrospective analysis of electronic health records (EHR), we sought to determine the degree to which self-reported race/ethnicity contributed to the probability of adverse drug reactions for these drugs. All models used individuals self-reporting as White as the comparison group. The majority of apparent associations between different racial groups and drug toxicity observed in the “race only” model failed to remain significant when we corrected for covariates. We did observe self-identified Asian race as a significant predictor (*p* = 0.016) for warfarin hemorrhagic events in all models. In addition, patients identifying as either Black/African-American (*p* = 0.001) or Other/Multiple race (*p* = 0.019) had a lower probability of reporting an adverse reaction than White individuals while on simvastatin even after correcting for other covariates. In both instances where race/ethnicity was predictive of drug toxicity (i.e., warfarin, simvastatin), the findings are consistent with the known global variability in the pharmacogenes described in the CPIC guidelines for these medications. These results confirm that the reliability of using self-identified race/ethnic information extracted from EHRs as a predictor of adverse drug reactions is likely limited to situations where the genes influencing drug toxicity display large, distinct ethnogeographic variability.

## 1. Introduction

While the U.S. Food and Drug Administration has added pharmacogenetic information to over 150 drug labels (https://www.fda.gov/drugs/science-and-research-drugs/table-pharmacogenomic-biomarkers-drug-labeling, accessed on 10 May 2021), the clinical implementation of this guidance is limited at best. As a result, the Clinical Pharmacogenetics Implementation Consortium (CPIC^®^) was created to establish peer-reviewed, evidence-based guidelines for utilizing the results from pharmacogenetic (PGx) tests. CPIC guidelines are conservative and aimed at providing a clinical decision framework for the utilization of PGx to guide pharmacotherapy for certain “priority” drugs. Among the prioritization of CPIC recommendations, drug/gene pairs with Level A notation indicate that “genetic information should be used to change prescribing of affected drug”. Hence, drugs with Level A evidence possess CPIC’s strongest prescribing recommendations.

There exists large disparities between various racial/ethnic/ancestral groups for both cerebrovascular/cardiovascular disease risk as well as response to pharmacotherapeutic interventions for these conditions [1,2,3,4,5]. Recent work in the field of pharmacogenomics has increasingly focused on the identification of ethnicity/population-specific variation in drug metabolizing and drug target genes that can help account for these differences. For example, individuals identifying as having African ancestry have a higher risk of developing cardiovascular disease (CVD) than individuals of European-American ancestry (EA) [1]. African-Americans (AAs) have a higher on-treatment platelet reactivity with antiplatelet drugs such as clopidogrel, suggesting a pharmacogenetic variation in drug metabolism [6]. Observations of differential racial or ethnic risk of disease/treatment failure is not only limited to thrombotic disease. For instance, Asian ancestry confers a higher risk for intracerebral hemorrhage (ICH) amongst all ethnicities worldwide. In the US, AAs also carry a greater risk of ICH than individuals of European ancestry [7].

There are three cardiovascular drugs that carry Level A prescribing guidance by CPIC: warfarin, clopidogrel and simvastatin. Warfarin is an oral anticoagulant that targets Vitamin K oxidoreductase complex 1 (VKORC1) thereby preventing the carboxylation/activation of clotting factors. A large amount of research supports variation in *CYP2C9* and *VKORC1* significantly contributing to both the warfarin dose and incidence of severe toxicity (i.e., intracerebral hemorrhage) [8]. Clopidogrel, a prodrug, is an anti-platelet agent that targets the P2Y12 adenosine receptor. CPIC guidance on clopidogrel focuses on the impact of *CYP2C19* variation and treatment response in patients with acute coronary syndrome (ACS) undergoing percutaneous coronary intervention (PCI). Simvastatin is an HMG-CoA reductase inhibitor used for the treatment of hypercholesterolemia. While generally well-tolerated, simvastatin use has been associated with myalgias, myopathy and, in rare cases, rhabdomyolysis [9,10]. While the frequency of reported statin-associated myalgias is no different than placebo [11,12], severe muscle toxicity has been associated with allelic variation in the *SLCO1B1 (OATP1B1)* gene [10] which facilitates hepatic uptake of the drug.

The use of race and ethnicity in medicine is controversial due to the large variability amongst different racial/ethnic groups, lack of detailed ethnic information, the co-existence of social and environmental determinants of health, and the broad geographic categorization of “racial” groups (i.e., Asian, African). Yet, race and ethnicity are still employed as surrogate biomarkers in clinical practice [13,14] including in many US FDA prescribing guidelines [15]. For each of the drugs described above, considerable variability amongst different ethnic/ancestral/racial populations [16,17,18,19,20] exists within their respective pharmacogenes covered in the CPIC guidelines. Given this variability, and its potential impact on drug response and toxicity, we sought to determine the degree to which race/ethnicity contributed to the frequency of adverse drug reactions for these priority drugs. We performed a retrospective analysis of electronic health records (EHR) for these drugs from a large, diverse medical center in Washington, DC.

## 2. Materials and Methods

### 2.1. Subject Recruitment

The study was considered exempt by the George Washington University Institutional Review Board (GWU IRB #NCR202660). We extracted all data from the GWA query that was developed to screen for patients at the George Washington University Medical Center Medical Faculty Associates EHR (Allscripts) using a drug-specific query. We extracted all data from the patients’ EHR. Inclusion criteria included in the study were >18 years of age and on at least one of the study drugs during the time period 2016–2020. For eligible subjects, we collected demographic information data (age, type of insurance if any, self-reported race and ethnicity), clinical data (BMI) and presence or absence of clinically significant drug interactions and outcomes (see Appendix A, Table A1).

### 2.2. Statistical Analyses

Inference was conducted independently for each of the warfarin, clopidogrel, and simvastatin patient samples. The warfarin and clopidogrel patient samples featured two primary endpoints of interest: any hemorrhaging events, and any adverse events (including hemorrhaging). The simvastatin sample only featured adverse outcomes, excluding hemorrhage, as an endpoint. For each sample and endpoint, an initial logistic regression featuring only race and ethnicity as predictors was fit to determine if there were any unadjusted differences in risk profiles between racial groups. After the baseline regressions were fit, a series of logistic regressions were conducted to account for other clinical covariates. These regressions featured main effect and interaction terms for race and ethnicity, as well as age, body mass index (BMI), and indicators for diabetes, hypertension, coronary artery disease, chronic obstructive pulmonary disease, high creatinine levels, high alanine aminotransferase (ALT) levels, other prescription drug use, alcohol use, and tobacco use. A series of prespecified tests were then conducted for each of these adjusted regressions to test for the importance of groupwise race and ethnicity interactions, high ALT values, and groupwise race and ethnicity effects. Due to missing patient data for the high creatinine and high ALT indicators, adjusted regressions were performed on ten imputed datasets for each sample, with imputation being performed through logistic regressions. Due to the imputation scheme, groupwise statistical tests were conducted using Rubin’s likelihood ratio test and Rubin’s Wald test, while individual predictors were assessed using Rubin’s Wald test.

## 3. Results

After adjusting for multiple entries for patients during the five-year period, we obtained 2139, 4190 and 11,522 records for warfarin, clopidogrel and simvastatin, respectively. The descriptive statistics for each drug group are provided in Table 1. Similar covariates were obtained for each population. The primary endpoints for warfarin and clopidogrel were total number of bleeding events and adverse drug reactions. For simvastatin, we explored the total number of reported adverse events as our primary clinical endpoint. This was inclusive of myopathic events which were found to be unrelated to simvastatin exposure (data not shown). The individual ICD-10 codes and list of clinically significant drug interactions included in our models are provided in Appendix A, Table A1. Importantly, we utilized racial/ethnic information exactly as provided in the electronic health record (EHR) for our analyses. All comparisons in the study were made within each drug group using the self-identified White population as the reference group for our analyses.

### 3.1. Warfarin

Warfarin has an extremely narrow therapeutic index and exhibits a large degree of heterogeneity for both treatment outcome and adverse events [8,21]. Bleeding/hemorrhagic events associated with suboptimal warfarin dosing are associated with significant morbidity and mortality [19,22,23]. We focused on comparing the frequency of both overall adverse events and hemorrhagic complications between different racial/ethnic groups compared to self-identified White patients. Co-variates for the warfarin group included hypertension (29.9%), diabetes (15.5%), coronary artery disease (CAD, 10%), impaired hepatic function (alanine aminotransferase (ALT); 3.1%), impaired renal function (creatinine clearance >1 mg/dL; 16.8%) and chronic obstructive pulmonary disease (COPD, 3.6%). Tobacco use and alcohol dependence/abuse was present in 2.4% and 3.6% of the subjects, respectively. Warfarin, a CYP2C9 substrate, has many potential drug interactions. Nearly 11% of subjects were prescribed medications with the potential of causing clinically significant interactions with warfarin. Almost a third of patients were also prescribed an NSAID according to the EHR (32.8%).

**Figure 1:**
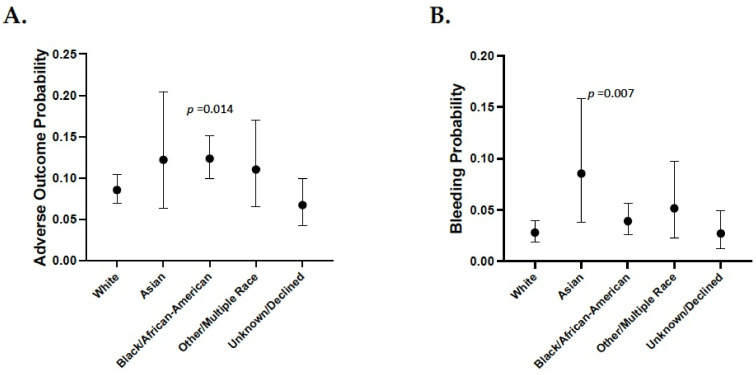
Race-only model for warfarin adverse events and bleeding events. Shown is the probability of experiencing any adverse outcome (**A**) or bleeding events (**B**) while taking warfarin based on race only. All comparisons used the White group as the reference.

The frequency of patients experiencing any adverse event was significantly higher (*p* = 0.014) for individuals identifying as Black/African-American (compared to White race) in the race-only model (Figure 1A). However, this difference disappeared when correcting for the covariates (Table 2). In the Asian group (3.8% of the group), the probability of experiencing any hemorrhagic complication while taking warfarin was significantly higher than the White group (*p* = 0.007) (Figure 1B). This risk persisted when correcting for all covariates (Table 3). Female gender (*p* = 0.022), diabetes (*p* = 0.019), coronary artery disease (*p* = 0.002), chronic obstructive pulmonary disease (*p* = 0.015) and concurrent NSAID usage (*p* = 0.001) were all marginally associated with a greater risk of warfarin adverse drug reactions. Hypertension and elevated creatinine levels were strongly associated with both warfarin adverse events as well as bleeding risk.

### 3.2. Clopidogrel

The study population was mostly Black/African-American (36.2%), followed by 35.3% of patients reporting as White, 13.4% identifying as Unknown/Declined, 9.0% identifying as Other/Multiple races and 6.2% identifying as Asian (Table 1). Clinically significant comorbidities for the clopidogrel population included hypertension (48.3%), coronary artery disease (CAD, 41.3%), diabetes (31.6%) and elevated creatinine (16.8%). Concurrent tobacco use and alcohol dependence/abuse was present for 4.7% and 3.7% of the subjects, respectively. We found that 27.8% of subjects had co-exposures to clinically significant interacting drugs and 17.7% were prescribed an NSAID.

**Figure 2:**
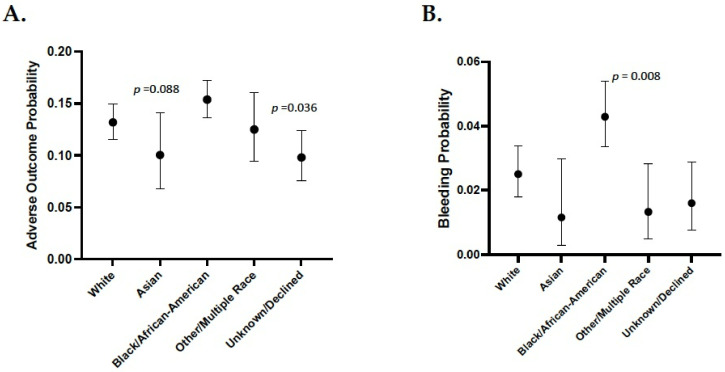
Race-only model for clopidogrel adverse events and bleeding events. Shown is the probability of experiencing any adverse outcome (**A**) or bleeding events (**B**) while taking clopidogrel based on race only. All comparisons used the White group as the reference.

Similar to warfarin, the most severe adverse outcome to clopidogrel is bleeding/hemorrhage events [20]. In addition, clopidogrel under-dosing has been associated with treatment failure related to *CYP2C19* metabolizer status [24,25]. The marginal probabilities of experiencing any adverse outcome was significantly higher in self-identified Black/African-American patients (*p* = 0.088) in the race only model (Figure 2A). Of note, respondents with an unknown race (*p* = 0.036) were significantly less likely to report an adverse event. Similar to warfarin, race was no longer a significant predictor after correcting for the covariates (Table 4). Factors associated with adverse events on clopidogrel included: hypertension (*p* = 0.015), tobacco usage (*p* = 0.021), elevated serum creatinine (*p* = 0.0003), NSAID usage (*p* = 0.001), concomitant NSAID usage (*p* = 0.001) and any drug interaction (*p* = 0.009). The latter predictor was insignificant when concomitant NSAID use was omitted from the model. In contrast, female gender (*p* = 0.006), diabetes (*p* = 0.014) and age (1.05 × 10^−6^) were less likely to report an adverse outcome with clopidogrel (Table 4). We found that patients reporting as Black/African-American exhibited a greater likelihood of experiencing a bleeding event (Figure 2B) which did not remain predictive when we corrected for other covariates (Table 5). In this cohort of patients, hypertension (*p* = 6.24 × 10^−5^), COPD (*p* = 0.022), tobacco use (*p* = 0.05), and elevated serum creatinine (*p* = 0.004) were associated with an increased risk of a bleeding event regardless of race/ethic group (Table 5). While not the focus of the current investigation, we found no reports of treatment failure (based on ICD-10 code) in the study population (data not shown).

### 3.3. Simvastatin

Given the broad indication of statins for the treatment of dyslipidemia, the greatest number of subjects (*n* = 11,522) were present in the simvastatin group. The population consisted of White (40.0%), Black/African-American (28.9%), Asian (7.0%), Unknown/Declined (14.0%) and Other/Multiple race (10.2%). (Table 1) The largest comorbidity for the simvastatin group was hypertension (38.2%). Other comorbid conditions included diabetes (26.2%), coronary artery disease (5.1%), elevated creatinine (11.3%) and alcohol use/dependence (4.1%). We found that 27.8% of subjects had been prescribed clinically significant interacting drugs.

**Figure 3:**
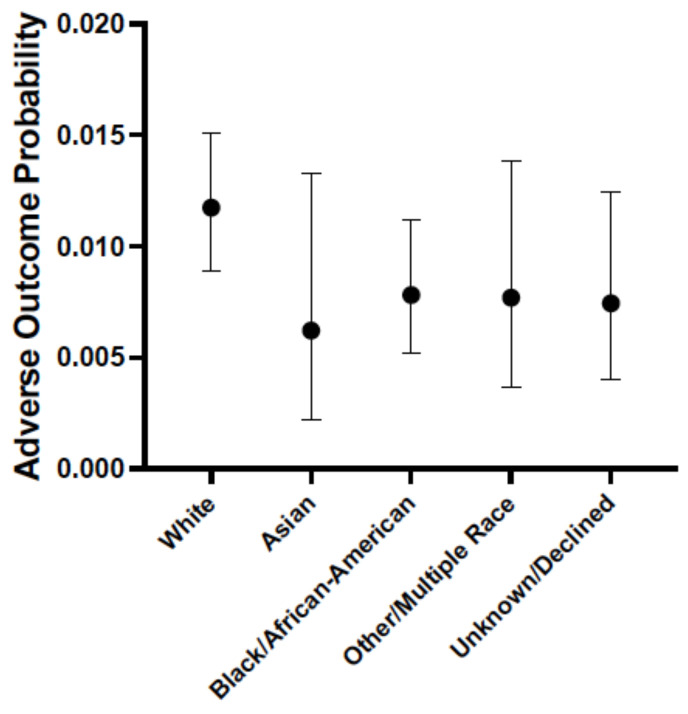
Race-only model for simvastatin adverse events. Shown is the probability of experiencing any adverse outcome while taking simvastatin based on race only. All comparisons used the White group as the reference.

The major adverse event for simvastatin intolerance is clinically significant myopathy and, in rare cases, rhabdomyolysis [9,10]. The former has been determined to be no greater than a placebo effect in the absence of elevated creatinine kinase levels [9,12]. However, either myopathy and/or rhabdomyolysis, in the presence of elevated enzyme levels, has been determined to be related to pharmacogenetic variation in the *SLOC1B1* gene [10]. When considering race alone, the probability of experiencing any adverse outcome while taking simvastatin was the same across all racial/ethnic groups (Figure 3). However, we found that being either of Black/African-American race (*p* = 0.001) or of Other/Multiple race (i.e., *p* = 0.019) was associated with a lower probability of reporting an adverse reaction while on simvastatin (Table 6) when correcting for all covariates. Furthermore, the only covariates that were significantly associated with an increased probability of adverse reactions were lower age (*p* = 0.00004) and having comorbid hypertension (*p* = 0.00003).

## 4. Discussion

Heterogeneity in drug response is a common issue in all areas of medicine. Although identification of factors accounting for this variability is far from complete, key pharmacogenetic factors that influence their efficacy and/or toxicity have been identified for many drugs. For those factors related to genetic variation, CPIC was established to provide evidence-based guidance to practitioners for certain drug:gene pairs (https://cpicpgx.org, accessed on 10 April 2021). Extensive genetic variability exists within the human population for most, if not all, of the pharmacogenes covered in the CPIC guidelines. This variability has in part contributed to the use of race and ethnicity in clinical prescribing guidelines [14]. Here, we have examined the degree to which self-identified race may contribute to the probability patients will experience adverse events with three cardiovascular drugs carrying the strongest prescribing recommendations by CPIC. The results suggest that apparent associations between race and adverse events is highly drug-specific and is oftentimes superseded by other clinical and environmental factors (i.e., clopidogrel). However, in a few instances self-reported race appears to be a significant predictor of drug toxicity in lieu of other covariates (i.e., warfarin bleeding risk in Asians and lower simvastatin adverse events in AA). In these situations, the associations are consistent with known heterogeneity for the relevant pharmacogenes covered in the CPIC guidelines.

The probability of experiencing any adverse drug reaction with warfarin was not associated with a specific race/ethnic group when corrected for comorbidities except in self-identified Asian patients. Consequently, the widespread use of race for the purpose of predicting adverse reactions to warfarin is not warranted based on our study. Predictably, we found that the known risk factors for warfarin toxicity (i.e., hypertension, CAD, COPD, renal impairment, and NSAID usage) [23], in addition to female gender, were significant predictors of warfarin adverse outcomes (Table 2). Diabetes (Type I or II) diagnosis was also associated with an increased probability of experiencing any adverse event while taking warfarin (Table 2). This is potentially related to either the significant pharmacokinetic interactions between warfarin and some antidiabetes drugs [26] and/or other comorbidities (i.e., hypertension) commonly present in diabetic patients. When we focused on the probability of experiencing any bleeding events while taking warfarin, we observed that hypertension and renal impairment both increased probability (Table 3) which is consistent with other findings [23]. The likelihood of having a bleeding event on warfarin was significantly higher in self-identified Asian individuals in both the race-only and full regression models (Figure 2, Table 3). A possible explanation for this finding might be related to the higher frequency of carriers of the *VKORC1* haplotypes which confer a low-dose warfarin phenotype in Asian populations [17,27] potentially increasing the risk of warfarin toxicity [19,28]. Although there is a large degree of ethnogeographic variation amongst different Asian populations [27], there is an overall greater number of “low dose” *VKORC1* genotypes in Asians compared to other populations [29]. For example, the frequency of the −1639 CC/CT (rs9923231) and 1173 GG/GT (rs9934438) alleles, which have lower warfarin dose requirements, are much higher in the Chinese vs. Caucasian populations [29]. Hence, apparent associations between self-identified Asian race and warfarin bleeding risk are plausible and consistent with the known pharmacogenetics of this drug.

Both the U.S. FDA [30] and CPIC [31] warn of therapeutic failure of clopidogrel for preventing thrombotic events in individuals who are *CYP219* poor metabolizers. There is considerable worldwide variability within the *CYP219* gene suggesting that certain ethnic or geographically distinct populations may potentially have high on-treatment platelet reactivity with this drug [32,33] or potentially greater risk of adverse events [33]. Similar to warfarin, we did not find that race was associated with any adverse events (Figure 2A; Black/African-American) or bleeding events (Figure 2B; Black/African-American, Other/Multiple race) while taking clopidogrel when corrected for covariates. (Table 4 and Table 5). Given that many clinical covariates already implicated in predicting clopidogrel response [32] were significantly predictive of both clopidogrel adverse events (Table 4) and bleeding events (Table 5), any apparent associations between race and drug toxicity are better explained by these factors in this study population. Lastly, the primary clinical endpoints of this study focused on adverse events and not treatment failure (i.e., thrombotic events). It is likely that race and ethnicity are not surrogates for any genetic factors that influence the likelihood of experiencing adverse events while taking clopidogrel.

While all statins carry a small risk of myopathy and elevations in creatinine kinase and transaminase levels, simvastatin still has a relatively favorable safety profile [9], which is consistent with the low percentage of adverse events (0.9%) observed in this investigation (Table 6). In contrast to both warfarin and clopidogrel, the race-only model for simvastatin failed to yield any associations with adverse events (Figure 3). However, patients identifying as Black/African-American or of Other/Multiple race were significantly less likely to report an adverse event (Table 6) compared to self-reported White individuals when considering all other covariates. To the best of our knowledge, this is the first study to report such an association. Given the relatively small number of adverse events, these results are somewhat surprising and likely benefited from the relatively large sample size (*n* = 11,522). The hepatic uptake of simvastatin is mediated by the organic anionic transporter SLCO1B1 and associations between lower activity *SLCO1B1* allelic variants and simvastatin myopathy has been observed [34]. Consequently, CPIC recommends either dose-reduction or switching to an alternative statin (i.e., pravastatin, rosuvastatin) for patients carrying decreased function *SLCO1B1* alleles [10]. Interestingly, the African population has a lower frequency of *SLCO1B1* alleles conferring lower transporter activity [16,34]. For example, the SLCO1B1*15 allele occurs in approximately 2.9% of Africans and 13% of Europeans and the SLCO1B1*5 allele (2.8% of Europeans) has not been detected in African populations [35]. Moreover, individuals of European ancestry have a greater area under the curve (AUC) and slower clearance for pravastatin than African-Americans [36]. While we have no information on *SLCO1B1* genotype in our study population, it is possible that individuals identifying as Black/African-American in this study were less likely to carry decreased functioning *SLCO1B1* alleles relative to the “White” patients taking simvastatin. However, it is equally possible that environmental/nongenetic factors can account for this discrepancy in adverse events. For example, Black/African-American and/or Other/Multiple race patients could have been less likely to report adverse reactions or seek medical care while taking simvastatin. Indeed, racial disparities in adverse drug reactions have been observed in other studies [5,28]. As a result, these findings require further detailed investigation.

This study has several strengths including its diverse study population (>50% of patients identified as non-White) and the relatively large sample size of each drug group. However, there are several limitations to the study that warrant discussion. First and foremost, we have relied on EHRs for all of our analyses. These are inherently incomplete and oftentimes lack detailed information. We also utilized ICD-10 codes for our endpoints, and many covariates, which are frequently nonspecific and somewhat subjective. In addition, many studies have highlighted the limited usefulness of social constructs such as race and ethnicity data in retrospective studies. Indeed, the self-reported racial and ethnic information present in the EHR lacks granular information on specific racial and ethnic subgroups that would have strengthened this investigation and the validity of its conclusions. Along these same lines, we were unable to obtain genotypic information on the relevant pharmacogenes for each drug which would have allowed analysis to include genetic predisposition in addition to race/ethnicity. Similarly, we lacked DNA/data on ancestry informative markers as a more accurate index of self-reported race and ethnicity which would have more accurately characterized the ethnogeographic composition of our study population. Consequently, the broad categorization of race/ethnicity in the EHR is somewhat reflected in the large variability of probabilities observed in the race-only models. Taken together, any of the observed correlations between self-reported race/ethnicity and adverse treatment outcome may not be applicable to other populations in more rural areas and/or datasets that contain more detailed ethnoracial information.

The use of race and ethnicity in clinical decision-making and in U.S. FDA package inserts are controversial given that they do not adequately reflect subgroup populations and are confounded by social determinants of health and health inequities. This being said, both terms are often used as surrogate genetic markers of disease risk and therapeutic outcome. This work highlights the somewhat limited biological value of utilizing race and ethnicity as predictors of drug toxicity. As we have shown, apparent associations between adverse drug outcomes and race can often be explained by other clinical (i.e., co-morbidities) and environmental (i.e., drug interactions, tobacco usage) factors. In certain situations where the variability of pharmacogenes is geographically diverse, race/ethnicity may be informative variables to include along with other factors commonly used in therapeutic decision-making (i.e., liver/kidney function, BMI) for predicting ADRs. While interesting, the results of this study require further prospective analyses in large, diverse populations accompanied by biological samples that will help yield genetic factors that can potentially account for the findings observed in this investigation.

## Figures and Tables

**Table 1:** Characteristics of study populations.

Variable	Warfarin	Clopidogrel	Simvastatin
N =	2139	4190	11,522
Age (y)	71.5 (15.1)	71.9 (12.3)	69.5 (12.3)
BMI	29.7 (7.7)	29.1 (6.4)	29.6 (6.6)
Male	60.1%	62.4%	50.2%
Female	39.9%	37.6%	49.8%
Race			
White/European-American	47.1%	35.3%	40.0%
Black/African-American	28.8%	36.2%	28.9%
Unknown/Declined Race	13.9%	13.4%	14.0%
Other/Multiple Race	6.4%	9.0%	10.2%
Asian	3.8%	6.2%	7.0%
Diabetes			
No	84.5%	68.5%	73.8%
Yes	15.5%	31.6%	26.2%
Hypertension			
No	70.1%	51.7%	61.8%
Yes	29.9%	48.3%	38.2%
CAD			
No	90.0%	58.7%	95.0%
Yes	10.0%	41.3%	5.1%
COPD			
No	96.4%	95.4%	97.9%
Yes	3.6%	4.6%	2.1%
Alcohol Abuse/Dependence			
No	96.4%	96.3%	95.9%
Yes	3.6%	3.7%	4.1%
Tobacco Use			
No	97.6%	95.3%	97.1%
Yes	2.4%	4.7%	2.9%
Creatinine (>1 mg/dL)			
No	83.2%	75.1%	88.7%
Yes	16.8%	24.9%	11.3%
ALT (>50)			
No	96.9%	96.4%	98.8%
Yes	3.1%	3.6%	1.2%
NSAID Use			
No	67.2%	82.3%	na
Yes	32.8%	17.7%	na
Any Drug Interaction			
No	89.2%	72.2%	73.8%
Yes	10.9%	27.8%	26.2%
Outcomes			
Hemorrhage/Bleeding Event			
No	96.5%	97.2%	na
Yes	3.5%	2.8%	na
Any Adverse Outcome			
No	90.3%	86.7%	99.1%
Yes	9.7%	13.3%	0.9%

Abbreviations: BMI-body mass index; COPD- chronic obstructive pulmonary disease, CAD-coronoary artery disease, ALT-alanine aminotransferase, NSAID- non-steroidal anti-inflammatory drug.

**Table 2:** Logistic regression model for any warfarin adverse drug reactions relative to White/European-American patients ^#^.

	Estimate	SE	Statistic	*p*-Value
Intercept	−3.388	0.631	−5.370	8.76 × 10^−8^
Asian	0.283	0.378	0.747	0.455
Black/African-American	−0.019	0.194	−0.097	0.922
Other Race ^a^	0.360	0.324	1.111	0.267
Unknown Race ^b^	−0.047	0.298	−0.160	0.873
Hispanic or Latino Ethnicity	−0.226	0.398	−0.566	0.571
Unknown Ethnicity	−0.330	0.186	−1.776	0.076
Female	0.369	0.160	2.300	0.022
Age	0.005	0.006	0.787	0.431
BMI	−0.009	0.011	−0.863	0.388
Diabetes	0.450	0.191	2.355	0.019
Hypertension	0.847	0.171	4.958	7.68 × 10^−7^
CAD	0.634	0.207	3.055	0.002
COPD	0.729	0.300	2.425	0.015
Alcohol	0.492	0.369	1.333	0.183
Tobacco	0.547	0.384	1.424	0.155
Creatinine (>1 mg/dL)	0.793	0.192	4.130	4.43 × 10^−5^
NSAID Usage	0.558	0.161	3.460	0.001
Any Drug Interaction	0.135	0.223	0.605	0.545

^#^ Patients self-identifying as White/European were used as the reference group for all comparisons. ^a^ Other race includes aggregates of racial groups with small numbers or those with multiple races/ethnicities indicated. ^b^ Unknown race includes patients who declined to identify with any racial group.

**Table 3:** Logistic regression model for hemorrhage associated with warfarin use relative to White/European-American patients ^#^.

	Estimate	SE	Statistic	*p*-Value
Intercept	−3.996	0.954	−4.190	2.91 × 10^−5^
Asian	1.120	0.463	2.421	0.016
Black/African-American	−0.083	0.314	−0.264	0.791
Other Race ^a^	0.778	0.467	1.668	0.096
Unknown Race ^b^	0.337	0.467	0.722	0.471
Female Gender	−0.146	0.257	−0.568	0.570
Hispanic or Latino Ethnicity	−0.554	0.649	−0.853	0.394
Unknown Ethnicity	−0.591	0.305	−1.937	0.053
Age	−0.005	0.009	−0.527	0.598
BMI	0.010	0.016	0.604	0.546
Diabetes	0.166	0.300	0.552	0.581
Hypertension	0.989	0.272	3.642	2.77 × 10^−4^
CAD	0.542	0.322	1.682	0.093
COPD	0.299	0.502	0.596	0.551
Alcohol	−0.222	0.739	−0.301	0.764
Tobacco	0.585	0.563	1.039	0.299
Creatinine (>1 mg/dL)	0.798	0.283	2.820	0.005
NSAID Usage	0.227	0.256	0.886	0.376
Any Drug Interaction	−0.030	0.366	−0.082	0.935

^#^ Patients self-identifying as White/European were used as the reference group for all comparisons. ^a^ Other race includes aggregates of racial groups with small numbers or those with multiple races/ethnicities indicated. ^b^ Unknown race includes patients who declined to identify with any racial group.

**Table 4:** Logistic regression model for adverse drug reactions associated with clopidogrel use relative to White/European-American patients ^#^.

	Estimate	SE	Statistic	*p*-Value
Intercept	−0.987	0.441	−2.238	0.025
Asian	−0.297	0.234	−1.269	0.204
Black/African-American	0.016	0.124	0.133	0.894
Other Race ^a^	−0.099	0.193	−0.514	0.607
Unknown Race ^b^	−0.099	0.185	−0.533	0.594
Hispanic or Latino Ethnicity	0.023	0.247	0.094	0.925
Unknown Ethnicity	−0.134	0.115	−1.168	0.243
Female	−0.300	0.110	−2.730	0.006
Age	−0.021	0.004	−4.890	1.05 × 10^−6^
BMI	−0.014	0.008	−1.775	0.076
Diabetes	−0.275	0.111	−2.466	0.014
Hypertension	0.269	0.111	2.434	0.015
CAD	1.471	0.110	13.407	0.00
COPD	0.314	0.196	1.603	0.109
Alcohol	0.073	0.248	0.296	0.767
Tobacco	0.432	0.188	2.304	0.021
Creatinine (>1 mg/dL)	0.443	0.119	3.713	0.0003
NSAID Usage	0.395	0.117	3.389	0.001
Any Drug Interactions	0.438	0.167	2.622	0.009

^#^ Patients self-identifying as White/European were used as the reference group for all comparisons. ^a^ Other race includes aggregates of racial groups with small numbers or those with multiple races/ethnicities indicated. ^b^ Unknown race includes patients who declined to identify with any racial group.

**Table 5:** Logistic regression model for hemorrhage on clopidogrel relative to White/European-American patients ^#^.

	Estimate	SE	Statistic	*p*-Value
Intercept	−3.936	0.898	−4.382	1.21 × 10^−5^
Asian	−0.768	0.612	−1.255	0.209
Black/African-American	0.185	0.239	0.777	0.437
Other Race ^a^	−0.767	0.519	−1.478	0.140
Unknown Race ^b^	−0.089	0.412	−0.215	0.830
Female	−0.228	0.210	−1.086	0.278
Hispanic/Latino Ethnicity	0.438	0.484	0.905	0.366
Unknown Ethnicity	−0.314	0.235	−1.336	0.182
Age	−0.006	0.009	−0.707	0.480
BMI	−0.011	0.015	−0.721	0.471
Diabetes	−0.091	0.207	−0.439	0.661
Hypertension	0.998	0.249	4.008	6.24 × 10^−5^
CAD	0.391	0.204	1.916	0.055
COPD	0.687	0.299	2.297	0.022
Alcohol	−0.117	0.491	−0.238	0.812
Tobacco	0.589	0.300	1.964	0.050
Creatinine (>1 mg/dL)	0.601	0.206	2.916	0.004
NSAID Usage	0.522	0.209	2.504	0.012
Any Drug Interaction	0.930	0.261	3.561	0.0004

^#^ Patients self-identifying as White/European were used as the reference group for all comparisons. ^a^ Other race includes aggregates of racial groups with small numbers or those with multiple races/ethnicities indicated. ^b^ Unknown race includes patients who declined to identify with any racial group.

**Table 6:** Logistic regression model for any simvastatin adverse drug reactions relative to White/European-American patients ^#^.

	Estimate	SE	Statistic	*p*-Value
Intercept	−2.761	0.774	−3.568	0.0004
Asian	−0.757	0.477	−1.586	0.113
Black/African-American	−0.915	0.265	−3.458	0.001
Other Race ^a^	−0.976	0.416	−2.348	0.019
Unknown Race ^b^	−0.547	0.364	−1.503	0.133
Female	0.054	0.203	0.267	0.789
Age	−0.034	0.008	−4.128	0.00004
BMI	0.008	0.014	0.560	0.576
Diabetes	0.144	0.230	0.625	0.532
Hypertension	0.937	0.224	4.192	0.00003
CAD	0.275	0.404	0.682	0.495
COPD	−0.905	1.012	−0.894	0.371
Alcohol	−0.458	0.592	−0.774	0.439
Tobacco	−0.503	0.721	−0.698	0.485
Creatinine (>1 mg/dL)	0.524	0.282	1.860	0.063
Any Drug Interaction	0.011	0.230	0.047	0.962

^#^ Patients self-identifying as White/European were used as the reference group for all comparisons. ^a^ Other race includes aggregates of racial groups with small numbers or those with multiple races/ethnicities indicated. ^b^ Unknown race includes patients who declined to identify with any racial group.

## Data Availability

The data are not publicly available due to the existence of protected health information in the dataset.

## Appendix A

**Table A1:** List of International Classification of Diseases, 10th Revision (ICD-10) codes for co-variates, drug interactions and clinical endpoints.

Drug	Diagnostic Codes	Diabetes	HTN	CAD	COPD	Alcohol Abuse/Dependence	Tobacco Use	Presence of Implants/Grafts	Interacting Medications	Bleeding Events/ Hemorrhage	Other Adverse Events/Treatment Failure
Warfarin	I48, G45, I63, I82, I26	E10, E11	I10, I11, I12, I13, I15, I16	I25	J44	F10	Z72.0	na	aspirin, diclofenac, amiodarone, clopidogrel, ibuprofen, indomethacin, ketoprofen, naproxen, sulindac, cimetidine, clofibrate, fluoxetine, primidone, rifamycin, carbamazepine, sulfinpyrazone, oxcarbazepine, phenytoin, valproic acid, fenofibrate, gemfibrozil, tamoxifen, zafirlukast, dabigatran, rivaroxaban, apixaban, edoxaban	D69.9, I61, I62, K92.2, K92.0, R04.9, R04.8, K92.1, R58, D69, I31.2, I60, I85.0, K25.0, K26.0, K27.0, K52.1, K62.5, K66.1, M25.0, N92.4, N93, R04.1	D72.819, R21, N14.4, K75.9, I96, I70.26, I73, A48.0,L88
Clopidogrel	I25, I70, I73, I63, G45	E10, E11	I10, I11. I12, I13, I15, I16	I25	J44	F10	Z72.0	Z95.5, Z95.818, Z95.820, Z95.828, Z95.9	cimetidine, fluoxetine, ticlopidine, fluconazole, ketoconazole, voriconazole, warfarin, rivaroxaban, apixaban, dabigatran, edoxaban, diclofenac, ketoprofen, ibuprofen, indomethacin, naproxen, sulindac, etravirine, felbamate, ticagrelor, prasugrel	D69.9, I61, I62, K92.2, K92.0, R04.9, R04.8, K92.1, R58, D69, I31.2, I60, I85.0, K25.0, K26.0, K27.0, K52.1, K62.5, K66.1, M25.0, N92.4, N93, R04.1, R31, S06.4, S06.5, S06.8	G44.4, G44.41, D61.1, D70.1, D69.59, T82.856, T82.855, T82.857, T82.858, T82.867, T82.868
Simvastatin	E78, I25, I70, I73	E10, E11	I10, I11, I12, I13 I15, I16	I25	J44	F10	Z72.0	na	voriconazole, itraconazole, gemfibrozil, carbamazepine, oxcarbazepine, primidone, phenytoin, amiodarone, fenofibrate, tamoxifen, valproic acid, zafirlukast, amlodipine, diltiazem	na	T88.6, T88.7, T50.905, T46.6, K85.3, T46, T78, T78.9, Y43, Y44, Y52, Y88, G72.0, G72.9, G72.89, R74.0, K71.1, K71.2, K71.6, K71.9, K72.0, K72.9

Abbreviations: HTN—hypertension, CAD—coronary artery disease, COPD—chronic obstructive pulmonary disease. A code marked with an asterisk (*) represents the symptoms or manifestations of a disease.
